# Cyclosporine as Therapy for Traumatic Brain Injury

**DOI:** 10.1007/s13311-023-01414-z

**Published:** 2023-08-10

**Authors:** Magnus J. Hansson, Eskil Elmér

**Affiliations:** 1 Abliva AB, Lund, Sweden; 2 Department of Clinical Sciences, Mitochondrial Medicine, Lund University, Lund, Sweden

**Keywords:** Biomarkers, Brain injuries, Traumatic, Clinical trials as topic, Cyclosporine, Diffuse axonal injury, Drug evaluation, Preclinical

## Abstract

Drug development in traumatic brain injury (TBI) has been impeded by the complexity and heterogeneity of the disease pathology, as well as limited understanding of the secondary injury cascade that follows the initial trauma. As a result, patients with TBI have an unmet need for effective pharmacological therapies. One promising drug candidate is cyclosporine, a polypeptide traditionally used to achieve immunosuppression in transplant recipients. Cyclosporine inhibits mitochondrial permeability transition, thereby reducing secondary brain injury, and has shown neuroprotective effects in multiple preclinical models of TBI. Moreover, the cyclosporine formulation NeuroSTAT^®^ displayed positive effects on injury biomarker levels in patients with severe TBI enrolled in the Phase Ib/IIa Copenhagen Head Injury Ciclosporin trial (NCT01825044). Future research on neuroprotective compounds such as cyclosporine should take advantage of recent advances in fluid-based biomarkers and neuroimaging to select patients with similar disease pathologies for clinical trials. This would increase statistical power and allow for more accurate assessment of long-term outcomes.

## Introduction

Traumatic brain injury (TBI) is a major cause of death and disability. Globally, more than 50 million people have a TBI each year [1]. The burden of mortality and morbidity that TBI imposes on society makes TBI a pressing public health problem.

TBI has been defined as an “alteration in brain function, or other evidence of brain pathology, caused by an external force” [2]. Depending on the nature of that external force, it can be broadly categorized as diffuse or focal. However, these categories simplify what is a complex disease process with diverse and overlapping injury subtypes [3]. The heterogeneity of disease pathology and clinical course has posed substantial challenges in the development of neuroprotective therapies for TBI, resulting in a lack of clinically proven pharmaceutical interventions.

Another barrier for TBI drug development is understanding and addressing the complexity of the secondary injury cascades that follows the initial injury. One example of such an injury cascade has been described for diffuse axonal injury (DAI), a common type of TBI. In DAI, neurons become sheared, stretched, or otherwise damaged as a result of high-velocity translational or rotational forces. Axons respond to these mechanical forces and secondary insults by undergoing Wallerian degeneration [4]. Membrane polarization and ion homeostasis are disrupted in affected neurons and oxidative stress increases. Neurofilaments become compacted and axonal transport is impaired as a result of the fracturing of microtubules. As a result of calcium overload and oxidative stress, common in several types of injuries, the mitochondrial permeability transition pore (mPTP) forms, increasing mitochondrial membrane permeability and ultimately leading to irreversible neuron damage and brain atrophy [5, 6]. If we can attenuate the secondary injury cascades following TBI, in the case of DAI by preventing the transition from reversible damage to axon disconnection, then we have an opportunity to limit the extent of neurodegeneration and morbidity following trauma [7].

Cyclosporine has been used for decades to achieve immunosuppression in organ transplant recipients. Indeed, its potent neuroprotective effects were discovered by accident while it was being used as an immunosuppressant to prevent hippocampal graft rejection in an experimental forebrain ischemia model [8, 9]. Since then, cyclosporine has demonstrated neuroprotection in a wide range of animal models of neurological injury and disease, an effect that has been linked to its ability to stabilize mitochondrial function by preventing mPTP formation [10–12]. Cyclosporine, analogs of cyclosporine, and genetic tools to modulate mPTP formation have been widely explored in experimental traumatic central nervous system injury models [13]. Here, we summarize the preclinical and clinical development of NeuroSTAT^®^ (also known as CicloMulsion^®^), a novel parenteral formulation of cyclosporine which was developed to avoid the risk of anaphylactic reactions associated with Kolliphor EL^®^, which is used as an excipient in other cyclosporine products such as Sandimmune^®^ [14]. NeuroSTAT^®^ has been recognized by regulatory authorities in the US and Europe in their granting of orphan drug designation for treatment of moderate to severe TBI (US Food and Drug Administration designation 10-3197, European Union designation EU/3/10/791).

Following a number of unsuccessful interventional clinical trials, large natural history initiatives over the past few decades have sought to improve the precision of TBI classification and diagnosis, standardize data collection, and develop and validate relevant clinical outcome measures [15–17]. The next era of therapeutic clinical trials will be able to utilize these efforts to better target defined subgroups of TBI patients with disease pathologies likely to benefit from the tested intervention. Specifically, the use of imaging and fluid-based biomarkers can aid in both the selection of patients with specific types of disease pathologies and the monitoring of pharmacodynamic effects of drugs.

In this article, we summarize the molecular basis for the neuroprotective effect of cyclosporine before reviewing the preclinical and clinical evidence supporting its use in TBI. Finally, we will look at the opportunities for continued development of neuroprotective compounds such as cyclosporine for TBI, given recent advances in biomarkers and imaging.

## Molecular and Cellular Effects of Cyclosporine

Cyclosporine is a cyclic polypeptide of 11 amino acids whose pharmacological targets are cyclophilins, a family of enzymes with diverse roles in cellular processes. Its current therapeutic uses in organ transplantation and autoimmune diseases are mediated by binding to cyclophilin A. This binding inhibits calcineurin activity in T-helper cells and prevents lymphokine release and T-cell proliferation in cell-mediated immune responses [18].

Cyclosporine and its analogs have displayed neuroprotective properties in several models of acute and chronic neurological disease. Its neuroprotective effect is considered to be predominantly mediated via inhibition of another cyclophilin, cyclophilin D, which regulates the mPTP [19]. Cyclosporine’s ability to inhibit activation of the mPTP has been demonstrated in both rodent and human brain mitochondria [20–22]. In TBI, inhibition of the mPTP by pharmacological treatments or genetic knockout of cyclophilin D decreases mitochondrial damage, lesion volume, intra-axonal cytoskeletal destruction, and brain injury biomarker levels (see Table 1).

**Table 1:** In vivo efficacy studies of cyclosporine

Model/species	Dose (mg/kg)/route/timing	Findings/conclusions
**Impact acceleration—histology, biochemistry**		
IA/rats [46]	10/IT/30 min before injury	CsA bolus before TBI preserved mitochondrial morphology and limited delayed axotomy and axonal damage
IA/rats [47]	10/IT/30 min before injury	CsA bolus before TBI decreased neurofilament compaction and cytoskeletal damage
IA/rats [48]	10/IT/30 min post-injury	CsA bolus after TBI decreased neurofilament compaction, calpain-induced axonal injury, and APP accumulation in damaged axons
IA + hypothermia/rats [49]	10/IT (infusion)/40 min post-injury	Significant reduction in APP accumulation in CsA + rapid rewarming group
IA/rats [23]	3, 10, 20, 30, 50/IV; 10/IT following injury (over 1 h by microsyringe infusion pump)	10 mg/kg IV CsA gave a lower brain concentration of CsA vs. 10 mg/kg IT. 10 mg/kg IV CsA gave the greatest reduction in injured axon density vs. controls and was significantly more effective than 20 or 30 mg/kg IV
IA/rats [50]	10/IT, 20/IV, 35/infusion 1 h 30 min post-injury	Significant neuroprotection with IT CsA (preservation of NAA and partial prevention of ATP loss). The 20 mg/kg IV dose failed to ameliorate biochemical damage; the 35 mg/kg dose showed 36% NAA recovery and 39% ATP restoration
**Behavioral**		
LFPI/rats [51]	10/IP/15 min post-injury + 28 daily boluses	Significant improvement in motor and sensorimotor functions. No beneficial effect on cognitive function
LFPI/rats [52]	0.625, 18.75/IV/30 min post-injury for 5 h	Both CsA doses abolished the 25% decrease in O_2_ consumption (VO_2_)
	0.375, 18.75/IV/infusion 60 min preinjury + 2 h post-injury	0.375 mg/kg CsA improved acute motor deficits. 0.375 and 18.75 mg/kg doses of CsA improved cognitive deficits.18.75 mg/kg worsened acute motor function
FPI/rats [26]	10, 20/IV/15 min and 24 h post-injury	FPI: 10 mg/kg dose gave histological protection (20 mg/kg worsened working memory) CCI: 20 mg/kg dose impaired Morris water maze performance; neither dose showed benefit for any outcome PBBI: no benefit for any outcome; mortality increased with the 20 mg/kg dose, partly due to the solvent vehicle. No positive effects on biomarker levels in any of the models
CCI/rats [26]		
PBBI/rats [26]		
IA/sheep[53]	10/IT/30 min post-injury	Reduction in APP mRNA 2 and 6 h post-injury
**Electrophysiological**		
Midline FPI/rats [54]	20/IP/15 min post-injury or 1 h post-injury	Administration at 15 min post-injury gave significant protection of the CAP area. Administration at 1 h did not significantly protect CAPs but was associated with atypical waveforms in N1 CAPs, including shorter CAPs and reduced refractoriness
**Cortical contusion injury—histology, mitochondrial function, imaging**		
CCI/rats and mice [55]	20, 40/IP/5 min before injury or 15 min post-injury + 24 h post-injury	All doses demonstrated a significant reduction in cortical damage in mice and rats. The lowest doses of CsA gave the greatest sparing of cortical tissue. No significant difference between pre- and post-treatment
	20, 40, 150/IP/5 min before injury (mice only)	
CCI/rats [56]	20/IP/15 min post-injury	Maintenance of mitochondrial transmembrane potential. Decrease in mPTP opening. Decrease in mitochondrial Ca^2+^. Decrease in ROS generation
CCI/rats [25]	20/IP/15 min post-injury followed by 0 or 20/IP/24 h or 4.5, 10/SC pump for 7 days	Significant reduction in lesion volume for all groups. The best results with 10 mg/kg/day continuous infusion for 7 days resulting in 74% reduction in lesion volume
CCI/rats [57]	20, 35/IV/1 h infusion 30 min post-injury	No significant change in brain water content and no exacerbation of brain edema with IV administration
CCI/rats [58]	20/IP/boluses 15 min post-injury + 24 h post-injury	CsA significantly decreased the extent of damage and C-tau levels in the ipsilateral hippocampus
CCI/rats [59]	20/IP/immediately post-injury	Significant decrease in Evans blue extravasation
CCI/mice [60]	20/IP/15 min post-injury	Significantly decreased protein nitration and lipid peroxidation in mitochondria 12 h after injury; reversal of decrease in RCR, but not the decrease in ETS capacity and complex II activity in mitochondria
CCI/rats [61]	20/IP + 10/SC per day through implanted pump for 72 h initiated 1, 3, 4, 5, 6, or 8 h post-injury	CsA treatment initiated at any of the post-injury times resulted in significantly less cortical damage. Treatment begun in the first 3 h was significantly more protective than that begun at 4 and 8 h
CCI/rats [62]	20/IP/15 min post-injury	Improvement of respiration at 24 h by CsA in the more severely impaired synaptic population of isolated mitochondria
CCI/rats [6]	20/IP/15 min post-injury + 10/SC infusion for 3 days	Significant reduction of 4-HNE binding to mitochondrial proteins at 72h by CsA, but no effect on α-spectrin degradation or respiration of isolated mitochondria
CCI/mice [63]	20/IP/15 min post-injury + 10/IP every 24 h for 5 days	Improvement of BBB stabilization and closure by CsA
CCI/rats [64]	20/IP immediately post-injury	Decrease of brain edema, lipid peroxidation, and ultrastructural neurodestruction by CsA
CCI/piglets [29, 30]	20/IV infusion for 5 days	CsA reduced the cortical and subcortical lesion volume by 35% and improved fractional anisotropy scores and MRS metabolite levels
**Behavioral + histology**		
CCI/rats [65]	20/IP/3 boluses 1 h post-injury + 24 h post-injury + 3 days post-injury	CsA had no significant effects on behavioral tests in rats
CCI/mice [66]	20/IP/15 min post-injury	Significant decrease in CSF levels of alpha-II-SBDPs
	20/IP/15 min post-injury + 24 h post-injury	Significantly improved motor function 48 h and 7 days post-injury. Significant decrease in the damaged area in the ipsilateral hemisphere 7 days post-injury
CCI/Mice [67]	20/IP/day or 1.26/IV/days for 7–14 days (lipoprotein biomimetic nanocarrier containing CsA)	Significantly reduced neuronal damage, astrogliosis, and microglial activation and improved Morris water maze performance
CCI/CypD-knockout mice [5]	20/IP/15 min post-injury + 24 h post-injury	CypD knockout improved mitochondrial bioenergetics, tissue sparing, and CA3 neuronal loss but not Morris water maze performance. CsA provided further tissue sparing in CypD-knockout mice
**Electrophysiological**		
CCI/rats [68]	20/IP/15 min post-injury	CsA prevented LTP impairment and LTD in CA1-CA3
**Aseptic cerebral injury**		
Aseptic cerebral injury/mice [69]	50/IP/2 h preinjury + 2 h post-injury	Decreases in density of apoptotic cells and size of injury (MRI). No significant change in neutrophil infiltration
**Combination studies and other models**		
CCI/rats [24]	1, 5, 10, 20, 40/IP boluses 15 min + 24 h post injury and 20/IP 15 min, 1 h, 6 h, or 24 h post injury + 20/IP after an additional 24 h	CCI: significant reduction in lesion volume for all groups. The best results with 20 mg/kg, which also showed effects at 6 and 24 h post-injury. Reduction of BBB disruption FPI: significant effect of both CsA groups combined compared to placebo
FPI/mice [24]	20, 40/IP boluses 15 min + 24 h post injury	
Rotating dental drill/rats (immature) [70]	20/IP/20 min post-injury + 24 h post-injury	CsA treatment significantly decreased brain weights, but similar effect from vehicle alone
CCI/rats (immature) [71]	20/IP/15 min post-injury	Both models: preserved mitochondrial bioenergetics and decreased hemispheric RCR differences Piglets: lactate:pyruvate ratio maintained, cerebral blood flow significantly increased, injured brain volume reduced by 42%
RNR/piglets [71]	2 × 20/IV/5 min post-injury + 12 h post-injury	
CCI/piglets [72]	10, 20, 40, 60 per day/IV/1 h, 6 h post-injury	CCI: 60 mg/kg at 1 h effective: ≥50% positive outcome rate (neuropathology plus mitochondrial function). 10, 20, and 60 mg/kg at 6 h effective: ≥45% positive outcome rate RNR: no CsA doses effective in the 1 h group. The 20 mg/kg dose in the 6 h group met effectiveness criteria (≥50% positive outcome rate)
RNR/piglets [72]	10, 20, 40, 60 per day/IV/1 h, 6 h post-injury	

*4-HNE* 4-hydroxynonenal, *APP* amyloid precursor protein, *ATP* adenosine triphosphate, *BBB* blood-brain barrier, *CAP* compound action potential, *CBF* cerebral blood flow, *CCI* controlled cortical impact, *CsA* cyclosporine, *CSF* cerebrospinal fluid, *CypD* cyclophilin D, *ELISA* enzyme-linked immunosorbent assay, *ETS* electron transport system, *FPI* fluid percussion injury, *IA* impact acceleration, *IP* intraperitoneal, *IT* intrathecal, *IV* intravenous, *LFPI* lateral fluid percussion injury, *LTD* long-term depression, *LTP* long-term potentiation, *mPTP* mitochondrial permeability transition pore, *MRI* magnetic resonance imaging, *MRS* magnetic resonance spectroscopy, *MWM* Morris water maze, *NAA N*-acetylaspartate, *PBBI* penetrating ballistic-like brain injury, *RCR* respiratory control ratio, *RNR* rapid nonimpact rotational, *ROS* reactive oxygen species, *SBDPs* spectrin breakdown products, *SC* subcutaneous, *TBI* traumatic brain injury

## Preclinical Development of Cyclosporine

Data from a wide range of in vivo models of TBI provide evidence of the neuroprotective effects of cyclosporine on mechanistic, histological, and behavioral endpoints (Table 1). More than 30 independent experimental studies of cyclosporine have been completed in different TBI models, including rodents and large animals. The neuroprotective effects of cyclosporine have been evaluated in impact acceleration animal models of DAI, as well as in models of primarily focal injury (cortical contusion) and experiments involving focal brain injury combined with DAI (fluid percussion models of TBI). Dosing regimens have varied from a single bolus to continuous infusions over several days, and the timepoint of evaluation following injury has varied from hours to weeks, depending on the outcome measure. A few studies have rigorously evaluated the optimal dosing regimen. For example, in a rat impact acceleration model, 10 mg/kg (infused post-injury over 1 h) was the most effective cyclosporine dose at attenuating axonal injury, as demonstrated by a 79% reduction in the mean density of damaged axons displaying amyloid precursor protein immunoreactivity at 24 h following injury [23]. In a series of papers evaluating the optimal dose and dosing regimen of cyclosporine following CCI injury in rats, an initial intraperitoneal loading dose of 20 mg/kg followed immediately by a continuous 7-day subcutaneous infusion of 10 mg/kg offered the greatest neuroprotective effect, as measured by a 74% reduction in lesion volume at day 7 [24, 25]. Overall, most in vivo studies have demonstrated beneficial effects of cyclosporine on the evaluated outcome measures. A few studies have found cyclosporine to be ineffective against TBI or to have negative effects (Table 1). Doses and dosing duration in relation to routes of administration have been discussed as a potential reason for these findings [13]. Moreover, in a study using a rat penetrating ballistic-like brain injury model, it was concluded that the vehicle for cyclosporine contributed to the observed negative effects on outcome measures [26]. However, the in vivo studies collectively demonstrate that cyclosporine exerts neuroprotective effects by (i) preserving mitochondrial function and morphology, (ii) maintaining axonal/cytoskeletal integrity, (iii) reducing the extent of proteolytic processes activated by or operant in TBI, (iv) minimizing the volume of the traumatic lesion, and (v) preserving peri-contusional viability.

### Piglet Study of NeuroSTAT

**Fig. 1:**
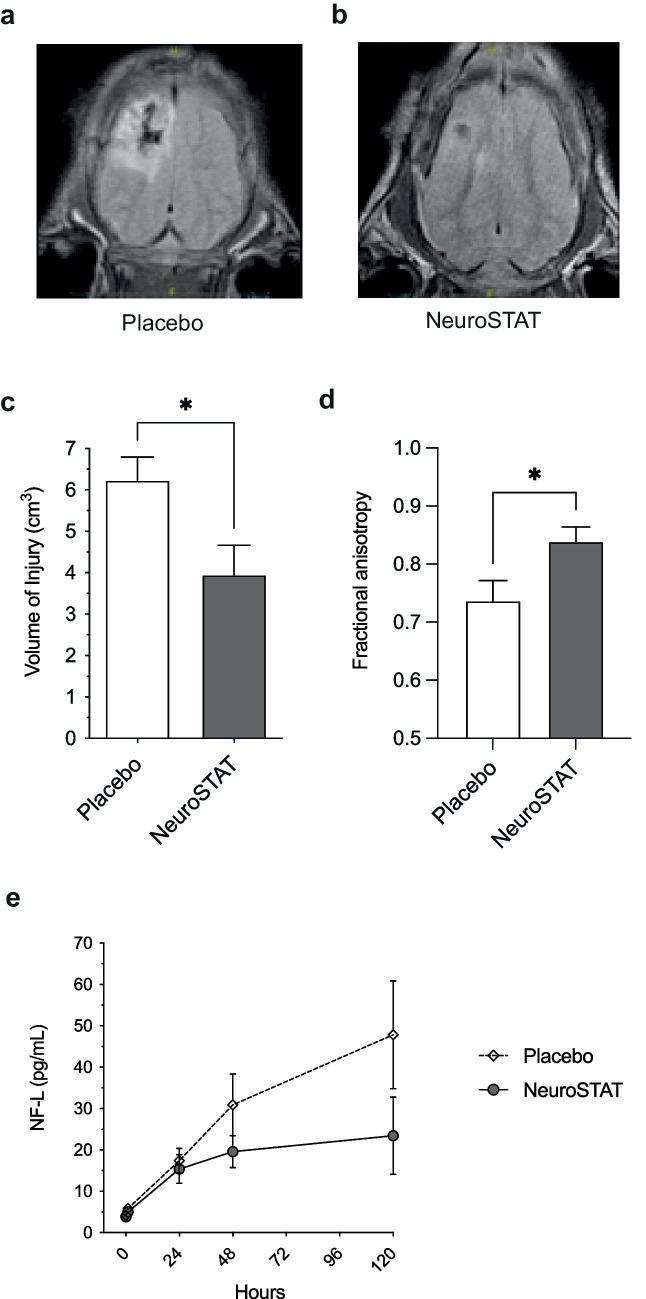
Translational efficacy outcomes of NeuroSTAT in a piglet study with controlled cortical impact injury. Neuroimaging on day 5 post-injury depicting **A** magnetic resonance imaging anatomical images representative of the median injury in the NeuroSTAT-treated group and **B** in the placebo group. **C** Volume of injury measured by manual tracing on each slice of area of increased signal abnormality on FLAIR imaging by board-certified neuroradiologist blinded to treatment group. **D** Fractional anisotropy in peri-contusional tissue using diffusion tensor imaging. **E** Neurofilament light (NF-L) in serum day 1–5 post-injury. Data are presented as mean and SEM. *p<0.05. Adapted from [29, 30] with permission from the publisher

As illustrated in Table 1, the therapeutic potential of cyclosporine in various formulations has been evaluated in several different TBI models, mostly in rodents. Compared to rodent models, piglet models are more relevant to humans because the gyrencephalic pig brain resembles the human brain more closely in anatomy, growth, and development than do the brains of rodents. The efficacy of the NeuroSTAT cyclosporine formulation has been evaluated in a randomized, fully blinded study using a piglet controlled cortical impact (CCI) TBI model. The piglet study aimed to investigate whether treatment with NeuroSTAT could influence the volume of parenchymal injury, as well as markers of neuronal integrity and axonal injury. Based on the evaluation of optimal dosing regimens in previous animal models ([24, 25], see discussion above) as well as preliminary signals from clinical studies ([27, 28], see below), it was decided to administer NeuroSTAT as a 5-day continuous infusion. Some of the translational outcome measures of the study are summarized in Fig. 1. Compared to placebo (*n*=13), NeuroSTAT (20 mg/kg/day, *n*=11) reduced the cortical plus subcortical lesion volume by 35% [29]. Axonal injury in the peri-contusional area was also reduced, as evidenced by significantly improved fractional anisotropy scores obtained by magnetic resonance diffusion tensor imaging [30]. Spectroscopic imaging of neuronal viability metabolites in brain regions comprising injured and normal parenchyma 5 days after CCI showed significantly higher mean levels of *N*-acetylaspartate, gamma-aminobutyric acid, phosphocreatine, and taurine in NeuroSTAT-treated animals compared to placebo-treated animals. Levels of neurofilament light (NF-L), a biomarker of axonal injury, also showed a trend of being lower in serum and cerebrospinal fluid (CSF) of NeuroSTAT-treated animals compared to placebo-treated animals. The study thus demonstrated that continuous infusion of NeuroSTAT for 5 days following CCI in piglets diminished secondary brain injury.

## Clinical Studies of Cyclosporine in TBI

Clinical studies of cyclosporine in TBI are summarized in Table 2. Commercial cyclosporine formulations are referred to by name; other formulations are collectively referred to as “cyclosporine.”

**Table 2:** Clinical studies with NeuroSTAT^®^ and other cyclosporine formulations in TBI

Study	Objective(s)	Study design	Population	Subjects exposed to cyclosporine/total subjects	Dose regimen(s)	Main findings
**Completed studies—bioequivalence to Sandimmune**						
PXL99752 (NCT01692834) [14]	Primary: to compare pharmacokinetics of a single dose of NeuroSTAT (test product) with Sandimmune (reference product) Secondary: to compare the tolerability profile of NeuroSTAT with Sandimmune	Phase I, single-blind, laboratory-blind, subject-blind, single-dose, randomized, two-period crossover study	Healthy volunteers	63/65	NeuroSTAT: 5 mg/kg IV infusion over 4 h Sandimmune: 5 mg/kg IV infusion over 4 h	NeuroSTAT and Sandimmune bioequivalent based on AUC_0-last_ and *C*_max_ values derived from blood samples. 2 SAEs (anaphylactoid reaction and anaphylactic reaction) after administration of Sandimmune, both of which led to study discontinuation. 3 additional subjects discontinued the study: 2 after receipt of NeuroSTAT with premedication and 1 after receipt of Sandimmune with premedication (premedication was introduced part-way through the study because of the SAEs described above).
**Completed studies (target indication)—TBI**						
CHIC study (NCT01825044) [28]	To investigate pharmacokinetics, safety, and effects on biomarkers of efficacy of NeuroSTAT in patients with severe TBI	Phase II, open-label, nonrandomized, interventional study	Adults requiring ICU admission with evidence of nonpenetrating severe TBI requiring EVD and ICP	16/16	NeuroSTAT 2.5 mg/kg IV followed by 5 mg/kg/day (cohort 1) or 10 mg/kg/day (cohort 2) infused for 5 days	PK analysis of CSF samples confirmed brain exposure to cyclosporine was dose-dependent. No deaths during the study. Distribution of AEs similar between cohorts. 7 severe AEs in the 10 mg/kg/day cohort (0 in the 5 mg/kg/day cohort). Levels of four biomarkers (GFAP, NF-L, Tau, and UCH-L1) tended to decrease during the 5-day treatment period and to increase after the end of treatment.
**Studies of cyclosporine in TBI with other formulations**						
Study by Young and colleagues (a) [73] (b) [27]	(a) To characterize cyclosporine PK parameters at an early stage following acute TBI (b) To establish safety of ciclosporin following acute TBI	Phase II, randomized, placebo-controlled dose-escalation study	Adults with acute severe TBI	(a) 24/30 (b) 32/40	Placebo (saline) or cyclosporine every12 h for 72 h I: 0.625 mg/kg II: 1.25 mg/kg III: 2.5 mg/kg IV every 12 h for 72 h IV: 2.5 mg/kg loading dose followed by 5.0 mg/kg by continuous IV for 72 h	GOS and GOSE scores did not differ significantly between placebo and cyclosporine. Patients treated by continuous infusion for 72 h had a higher probability of a favorable functional outcome than patients who received separate infusions every 12 h. Mortality and incidence of SAEs not significantly different between cyclosporine and placebo. Percentages of patients with infections and abnormal liver function tests comparable between cyclosporine and placebo.
Study by Bullock and colleagues (a) [74] (b) [31, 32, 75]	(a) To detect the early effect of severe TBI upon cell-mediated immunity following treatment with cyclosporine (b) To evaluate the effect of cyclosporine on brain energy metabolism and cerebral hemodynamics To assess the safety, tolerability, and PK of cyclosporine	Phase II, prospective, randomized, placebo-controlled pilot study	Adults with severe TBI	(a) 36/49 (b) 37/50 (overlapping or partly overlapping with the patients reported in [74])	(a) No treatment (*n*=3), placebo (*n*=10), cyclosporine 5 mg/kg IV over 24 h (*n*=28) or cyclosporine 5 mg/kg/day IV over 48 h plus 250 mg ketoconazole (*n*=8) (b) Placebo (5% DW: *n*=13) or cyclosporine 5 mg/kg IV over 24 h (*n*=37)	T cell counts and the incidence of infection did not differ significantly between placebo- and cyclosporine-treated patients. Brain extracellular fluid glucose, lactate, and pyruvate levels significantly higher in patients treated with cyclosporine than in those who received placebo, whereas lactate/pyruvate ratio and glutamate levels were lower at various days after treatment in those receiving cyclosporine. MAP and CPP higher in the cyclosporine group than the placebo group throughout the 7-day monitoring period but did not exceed normal physiological ranges. Mean BUN levels higher for cyclosporine than for placebo at 24 and 48 h, but still within the normal range. Mean WBC count higher for cyclosporine than for placebo at 24 h Otherwise, no significant differences in laboratory test results between the two treatments. Incidence of AEs generally comparable between treatments. No significant treatment difference in neurological outcome based on GOS scores at 3 or 6 months.
Aminmansour et al., 2014 [33]	To evaluate the efficacy of cyclosporine in improving consciousness and cognitive dysfunction of patients with DAI after TBI To evaluate the safety of cyclosporine	Nonrandomized double-blind placebo-controlled study	Patients with TBI (GCS ≤10)	50/100	Cyclosporine 5 mg/kg, infused over 24 h starting within 8 h after trauma (*n*=50) Vehicle (5% DW) in the same time course (*n*=50)	No significant difference between treatment groups in GOSE scores or MMSE results after 3 and 6 months (all patients had moderate or severe impairment based on MMSE results). No meaningful differences in safety outcomes between treatment groups.

*AE* adverse event, *BUN* blood urea nitrogen, *CHIC* Copenhagen Head Injury Ciclosporin, *CPP* cerebral perfusion pressure, *DAI* diffuse axonal injury, *DW* dextrose water, *EVD* external ventricular drainage, *GFAP* glial fibrillary acidic protein, *GCS* Glasgow Coma Scale, *GOS* Glasgow Outcome Scale, *GOSE* Glasgow Outcome Scale-Extended, *ICP* intracranial pressure, *ICU* intensive care unit, *IV* intravenous, *MAP* mean arterial pressure, *MMSE* mini-mental state examination, *N-FL* neurofilament light, *PCI* percutaneous coronary intervention, *PK* pharmacokinetics, *SAE* serious adverse event, *TBI* traumatic brain injury, *UCH* ubiquitin carboxy-terminal hydrolase, *WBC* white blood cell

### Early Randomized Controlled Trials

In a randomized, double-blind trial of cyclosporine by Young and colleagues, patients with severe TBI (Glasgow Coma Scale [GCS] 4–8) were assigned to one of four dose cohorts (placebo *n*=2, cyclosporine *n*=8 for each cohort): 0.625, 1.25, or 2.5 mg/kg as a 2-h infusion every 12 h (6 doses) or a 2.5 mg/kg loading dose followed by 5 mg/kg/day as a continuous 72-h infusion. Efficacy was assessed at 3 and 6 months. Although Glasgow Outcome Scale (GOS) and Glasgow Outcome Scale-Extended (GOSE) scores did not differ significantly between placebo- and cyclosporine-treated patients, cyclosporine showed trends of improved functional outcomes across different dose cohorts. Outcome scores improved from poor to good at the 6-month assessment in 35% of cyclosporine-treated patients and 0% of placebo-treated patients. Moreover, patients treated by continuous infusion for 72 h had a higher probability of a favorable functional outcome than patients who received separate infusions every 12 h. The incidence of mortality and serious adverse events (SAEs) did not differ significantly between cyclosporine and placebo. Percentages of patients with infections and abnormal liver function test results were also comparable between treatments [27].

In another randomized, double-blind trial conducted by Bullock and colleagues, patients with severe TBI (GCS 3–8) were randomly assigned 3:1 to cyclosporine 5 mg/kg or placebo. Treatment was administered by continuous infusion over 24 h. Brain extracellular fluid levels of glucose, lactate, and pyruvate were significantly higher in patients treated with cyclosporine than in those who received placebo. Conversely, glutamate levels were significantly higher in the placebo group than the cyclosporine group 1 to 2 days after the end of study drug infusion, and lactate/pyruvate ratio was significantly higher in the placebo group 2 to 3 days after the end of study drug infusion. Cyclosporine administration was associated with increases in mean arterial pressure (MAP) and cerebral perfusion pressure (CPP) [31]. Moreover, MAP and CPP were significantly higher in the cyclosporine group than the placebo group from 0 through 3 days, although MAP remained within the normal physiologic range. Mean blood urea nitrogen (BUN) levels were higher for cyclosporine than for placebo at 24 and 48 h, but they too remained within the normal range. The mean white blood cell count was higher for cyclosporine than for placebo at 24 h. Otherwise, there were no significant differences in laboratory test results between the two treatments, and the incidence of adverse events (AEs) was generally comparable between treatments. No significant treatment difference in neurological outcome based on GOS scores was observed at 3 or 6 months [32].

### Nonrandomized Study

A nonrandomized, placebo-controlled clinical study in Iran tested cyclosporine on TBI patients with a GCS score of ≤10 and clinical and radiological evidence of DAI. Cyclosporine (5 mg/kg) or vehicle (5% dextrose water) was administered as a continuous 24-h infusion, starting within 8 h after trauma. Efficacy was assessed at 3 and 6 months. All patients in both the cyclosporine arm (*n*=50) and the placebo arm (*n*=50) had moderate or severe cognitive impairment at 3 and 6 months based on mini-mental state examination (MMSE) results [33]. There were no significant differences between treatment arms in MMSE results or GOSE scores at 3 and 6 months. While not presented in detail, data for deaths and infections were reported to be comparable between the cyclosporine and placebo arms. BUN levels at 48 h were higher in the cyclosporine arm than in the placebo arm. Otherwise, levels of other safety markers (creatinine, aspartate aminotransferase, alanine aminotransferase, and alkaline phosphatase) were within normal ranges and were comparable between treatment arms.

## Clinical Development of NeuroSTAT

### Bioequivalence Study

The first clinical study in the clinical development of NeuroSTAT was a Phase I open-label laboratory-blind crossover study in healthy volunteers to establish bioequivalence between NeuroSTAT and the reference cyclosporine formulation Sandimmune. NeuroSTAT and Sandimmune were administered as a single 5 mg/kg dose, infused at a constant rate over 4 h. Sixty-five subjects were enrolled in the study of whom 63 received at least one dose of study drug. The analysis included data for the 52 subjects who completed the study. For both AUC_0-last_ and *C*_max_, the 90% confidence intervals of the geometric mean ratios for NeuroSTAT/Sandimmune were within the bioequivalence range of 0.80–1.25 [14]. Two SAEs were recorded (anaphylactoid reaction and anaphylactic reaction), both after administration of Sandimmune. No SAEs were recorded after administration of NeuroSTAT.

### Copenhagen Head Injury Ciclosporin Trial

CHIC (Copenhagen Head Injury Ciclosporin) was an open-label, Phase Ib/IIa clinical trial that investigated the effects of two NeuroSTAT dosing regimens on pharmacokinetics, safety, and biomarkers of efficacy in patients with severe TBI (GCS 4–8) and clinical indication for external ventricular drainage and intracranial pressure monitoring. Patients were given a 2.5 mg/kg IV loading dose followed by a 5-day continuous infusion of 5 mg/kg/day (*n*=10) or 10 mg/kg/day (*n*=6). CSF cyclosporine concentration-time profiles showed that all patients had detectable cyclosporine concentrations. The PK profiles in blood and CSF were similar after the constant infusion was stopped, suggesting that elimination of CSF is determined by elimination in blood. The mean steady-state concentration indicated a dose-proportional exposure in CSF.

**Fig. 2:**
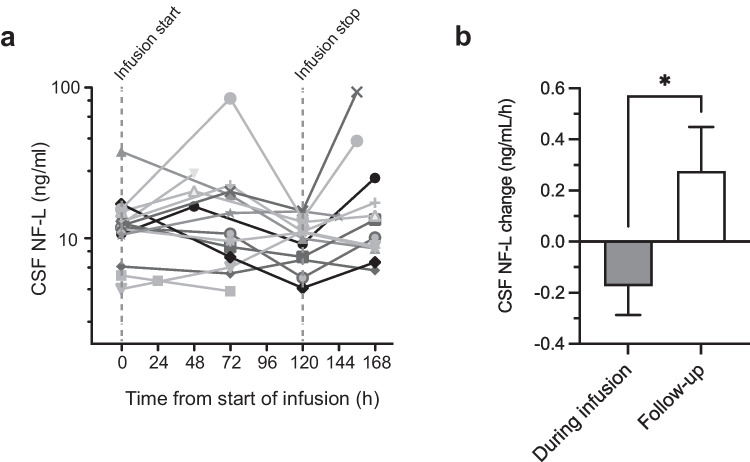
Pharmacodynamic signal of NeuroSTAT in the Copenhagen Head Injury Ciclosporin Phase Ib/IIa clinical trial. Temporal profile of brain injury biomarker neurofilament light (NF-L) in cerebrospinal fluid (CSF). **A** Individual levels of NF-L in CSF samples drawn at predose, during the continuous NeuroSTAT infusion, and after treatment had ended. Dashed vertical lines indicate the start and stop of infusion. CSF, cerebrospinal fluid. **B** Slopes of NF-L change during NeuroSTAT infusion and during follow-up after infusion stop. Data are presented as mean and SEM. *p<0.05. Adapted from [28] with permission from the publisher

A positive pharmacodynamic signal was detected in biomarkers measured in CSF samples. Levels of glial fibrillary acidic protein (GFAP), NF-L, Tau, and ubiquitin carboxy-terminal hydrolase (UCH)-L1 tended to decrease during the 5-day NeuroSTAT treatment period and to increase in the follow-up period after the end of treatment. Figure 2A illustrates by-patient CSF NF-L values over time. The shifts in trends for biomarker concentrations (slope after vs. during infusion) were statistically significant for NF-L (Fig. 2B) and the other investigated biomarkers [28]. These findings are exciting but should be considered preliminary given the lack of a control group and the small sample size.

Twenty-one AEs related to NeuroSTAT were reported in the CHIC trial. These included five events of increased plasma cystatin C, three events of increased plasma creatinine, and two events of oliguria. Plasma bilirubin levels increased during treatment in both dose groups, but the increase was more pronounced in the higher dose group (10 mg/kg/day). Moreover, compared to the 5 mg/kg/day group, hyperbilirubinemia was more frequent in the 10 mg/kg/day dose group. One patient in the 10 mg/kg/day group suffered from an SAE of acute renal tubular necrosis, likely as a result of concurrent sepsis. Four events of increased intracranial pressure were assessed as nonrelated to study treatment, and intracranial pressure was not obviously affected by NeuroSTAT administration.

## Future Directions

As outlined above, the broad neuroprotective effects of cyclosporine have been demonstrated in multiple preclinical studies with solid experimental evidence supporting cyclosporine’s efficacy in both diffuse and focal contusional injury models (Table 1). In addition, safe and tolerable dosing regimens have been explored in clinical studies of patients with severe TBI with mixed underlying pathology. Based on this exciting preclinical and clinical data, cyclosporine warrants further investigation as a potential therapy for TBI.

Recent advancements in the understanding of the biology of TBI, diagnostic and prognostic biomarkers, and medical imaging will facilitate further development of neuroprotective compounds. First, focusing on a single pathological TBI endophenotype (i.e., an internal measurable phenotype underlying a more complex phenotype [34, 35]) will minimize patient heterogeneity and increase the ability to assess pharmacodynamic outcomes. One appealing candidate here is a predominantly DAI endophenotype. DAI is one of the most common and severe forms of TBI, with a high degree of intracellular secondary injury cascade activation [3]. A systematic review and meta-analysis concluded that TBI patients with DAI have a three times higher risk of an unfavorable outcome than TBI patients without DAI [36]. The mechanism of action of cyclosporine is highly relevant to DAI in that prompt treatment of the primary injury can prevent subsequent brain atrophy.

Fluid biomarkers and neuroimaging are potentially sensitive measures for diagnosing DAI and tracking neurodegeneration following DAI [37]. Both have been included in large natural history studies, providing a valuable data source for adequate powering of investigational studies and for analyzing the associations between biomarker trajectories and long-term clinical outcomes [17, 38].

A biomarker of particular interest for DAI is NF-L, a protein found in long myelinated white matter axons. Studies have shown that serum NF-L increases as a result of TBI and that serum NF-L levels after TBI can predict clinical outcome [39–41]. Both the preclinical piglet study with NeuroSTAT and the clinical CHIC study demonstrated that NF-L levels were attenuated during cyclosporine therapy [28, 30].

Brain atrophy after TBI has been shown to be predictive of cognitive and neuropsychological outcomes [42–44]. Global and regional atrophy can be measured by repeat magnetic resonance imaging (MRI) scans, comparing a pseudo-baseline assessment soon after trauma to later follow-up assessments. MRI with diffusion tensor imaging also enables a sensitive assessment of DAI, and the location and extent of DAI as assessed by fractional anisotropy has been shown to predict the degree of progressive atrophy [45]. Advanced imaging techniques such as these should help clinical researchers to more accurately predict long-term outcomes in TBI patients based on short-term responses to cyclosporine.

## Conclusions

Since its serendipitous discovery, the neuroprotective effect of cyclosporine has been explored and validated in an extensive set of preclinical and clinical studies. Future clinical development of cyclosporine will be aided by an increased understanding of TBI endophenotypes and the further development of biomarkers and MRI as clinical research tools. These important advancements should increase the probability of success in clinical trials, enabling the evaluation of cyclosporine in a more homogenous population where clinical efficacy can be visualized and quantified. The time is now for the progression of neuroprotective agents for the treatment of TBI to address the unmet needs of patients with TBI who have been historically underserved.
